# Serum neuronal, glial and mitochondrial markers in autosomal dominant optic atrophy and Leber hereditary optic neuropathy

**DOI:** 10.1093/braincomms/fcaf446

**Published:** 2025-11-17

**Authors:** Alessandra Rufa, Domenico Plantone, Alessia Bargagli, Delia Righi, Tommaso Bacci, Valeria Serchi, Guido Primiano, Gian Nicola Gallus, Diego Lopergolo, Elena Pretegiani, Francesca Rosini, Sara Locci, Gian Marco Tosi, Nicola De Stefano

**Affiliations:** Neurology, Department of Medicine, Surgery and Neuroscience, University of Siena, 53100 Siena, Italy; EVAlab, Neurology, Department of Medicine, Surgery and Neuroscience, University of Siena, 53100 Siena, Italy; Neurology, Department of Medicine, Surgery and Neuroscience, University of Siena, 53100 Siena, Italy; Neurology, Department of Medicine, Surgery and Neuroscience, University of Siena, 53100 Siena, Italy; EVAlab, Neurology, Department of Medicine, Surgery and Neuroscience, University of Siena, 53100 Siena, Italy; Neurology, Department of Medicine, Surgery and Neuroscience, University of Siena, 53100 Siena, Italy; Ophthalmology, Department of Medicine, Surgery and Neuroscience, University of Siena, 53100 Siena, Italy; EVAlab, Neurology, Department of Medicine, Surgery and Neuroscience, University of Siena, 53100 Siena, Italy; Dipartimento di Neuroscienze, Organi di Senso e Torace, Fondazione Policlinico Universitario Agostino Gemelli IRCCS, 00168 Rome, Italy; Dipartimento di Neuroscienze, Università Cattolica del Sacro Cuore, 00168 Rome, Italy; Neurology, Department of Medicine, Surgery and Neuroscience, University of Siena, 53100 Siena, Italy; Neurology, Department of Medicine, Surgery and Neuroscience, University of Siena, 53100 Siena, Italy; EVAlab, Neurology, Department of Medicine, Surgery and Neuroscience, University of Siena, 53100 Siena, Italy; Department of Clinical Neurosciences, Centre Hospitalier Universitaire Vaudois (CHUV), 1005 Lausanne, Switzerland; EVAlab, Neurology, Department of Medicine, Surgery and Neuroscience, University of Siena, 53100 Siena, Italy; Stroke Unit, Department of Emergency-Urgency and Transplants, Azienda Ospedaliera Universitaria Senese, 53100 Siena, Italy; Neurology, Department of Medicine, Surgery and Neuroscience, University of Siena, 53100 Siena, Italy; Ophthalmology, Department of Medicine, Surgery and Neuroscience, University of Siena, 53100 Siena, Italy; Neurology, Department of Medicine, Surgery and Neuroscience, University of Siena, 53100 Siena, Italy

**Keywords:** mitochondria, neurodegeneration, Glia, LHON, ADOA

## Abstract

Leber hereditary optic neuropathy (LHON) and autosomal-dominant optic atrophy (ADOA) are the two most prevailing primary mitochondrial optic neuropathies. Both diseases preferentially affect the smallest retinal ganglion cells (GCs) of the papillomacular bundle, causing central visual loss in young patients. Although ADOA and LHON show striking similarities, including the convergence of underlying pathologic mitochondrial mechanisms, they differ clinically. The major distinction lies in the timing and progression of axonal damage during neurodegeneration. The exact reasons for these differences remain unclear, but they may, in part, be due to distinct patterns of mitochondrial dysfunction.

To identify differences that could point to distinct degenerative processes, we investigated clinical features, optical coherence tomography (OCT) findings, laboratory biomarkers [serum neurofilaments light chain (sNfL), serum glial fibrillary acidic protein (sGFAP) and serum growth differentiation factor-15 (sGDF15)] in a cohort of patients with these two heritable optic neuropathies in the chronic phase. Our OCT analysis reveals a more profound GC layer and papillomacular bundle loss in LHON, whereas ADOA shows a sparser damage of the retinal nerve fibre layer, including fibres originating from the nasal retina.

We also observed increased plasma levels of sNfL and GFAP in both groups, supporting the presence of ongoing neurodegeneration in both optic neuropathies.

Finally, our findings suggest the retinal astrocytes may play a contributive role in the neurodegenerative process at the level of the optic nerve head, particularly in ADOA.

## Teaser

Serum glial fibrillary acidic protein, neurofilament light chain and Growth/differentiation factor-15s were increased in the chronic stage of Leber hereditary optic neuropathy and autosomal-dominant optic atrophy, suggesting an ongoing neurodegeneration in both optic neuropathies with a role for optic nerve head astrocytes in this process.

## Graphical Abstract

**Graphical Abstract:**
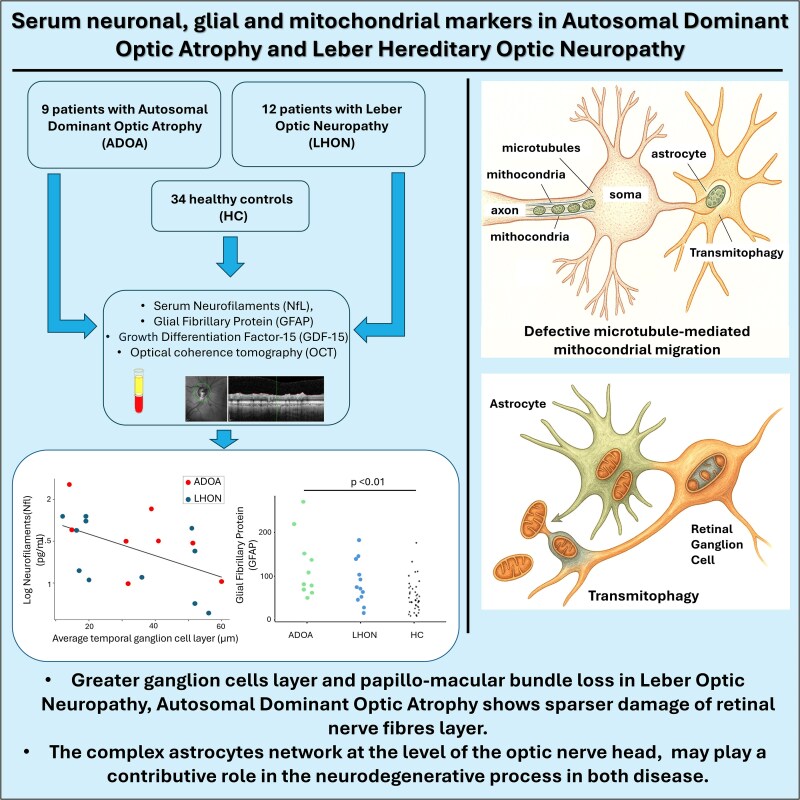

## Introduction

Leber hereditary optic neuropathy (LHON) and autosomal-dominant optic atrophy (ADOA), the prevailing hereditary optic neuropathies, are related to mitochondrial dysfunctions leading to death of the smallest-size retinal ganglion cells (GCs), a category of cells belonging to the parvocellular system with distinctive anatomical configuration and metabolic demands, making them particularly vulnerable to mitochondrial injury.^1,2^ Small GCs axonal loss in the papillomacular bundle (PMB) causes the typically observed impairment of the central vision and red/green hue channel.^3-5^

Clinically, ADOA and LHON share striking similarities, including the possibility of multisystemic manifestations, but they also show differences such as symptoms onset and progression, and subtle morphological and functional aspects of the optic nerve damage, that could indicate a difference in neurodegenerative process.

What accounts for these differences is not fully understood, but it could be partly explained by dissimilar mitochondrial dysfunctions.^6^

A distinct involvement of mitochondria in the pathogenesis of LHON and ADOA may arise from the underlying genetic mechanisms: most ADOA-associated variants are nuclear-encoded, with different effects and penetrance compared to the classical LHON variants, which are located in the mitochondrial DNA.

LHON, indeed, represents the prototype of a massive mitochondrial failure, characterized by a sudden collapse of ATP energy supply and a subsequent wave of retinal GC death. It is caused by three major mitochondrial DNA variants m.3460G>A, m.11778G>A, and m.14484T>C affecting, respectively, the *MT-ND1*, *MT-ND4* and *MT-ND6* genes that encode essential subunits of the mitochondrial respiratory chain complex I.^7^ Dysfunction of complex I affects the flux of electrons along the mitochondrial respiratory chain resulting in impaired oxidative phosphorylation (OXPHOS) and increased levels of reactive oxygen species (ROS).^8^

On the other side, ADOA is an example of the slowly progressive inability for the mitochondrial maintenance to preserve its efficiency. About 70% of individuals affected by ADOA carry pathogenic variants within the nuclear *OPA1* gene located at the *3q29* locus.^9-11^

The *OPA1* gene product is a *dynamin-like* GTPase protein located in the inner mitochondrial membrane (IMM).^9^ Together with *mitofusin 1 and 2* positioned in the outer membrane, it promotes the mitochondrial fusion which is significantly involved in various aspects of mitochondrial maintenance, including regulation of dynamics, preservation of the IMM stability and respiratory chain complexes, sequestration of the pro-apoptotic cytochrome c within the cristae and mitochondrial biogenesis and turnover.^12,13^

Furthermore, the onset of LHON is often associated with metabolic stressors, suggesting a greater sensitivity to environmental influences, whereas ADOA appears to be less affected by such external factors.

However, this might not be the full picture, and the potential influence of additional factors remains to be elucidated. In recent years, a crucial role of the glia in modulating the neurodegenerative process has started to be acknowledged.

Neurofilaments (NfLs) and glial fibrillary acidic protein (GFAP) are intracellular proteins released in the spinal fluid or peripheral serum following neuro-axonal and glial injury. Small fluctuations in their concentration can now be detected by ultrasensitive digital methods of immunoassay, offering insight about the progression of neuro-axonal and glial damage.^14-16^

Additionaly, mitochondrial dysfunction can be indirectly evaluated by assessing, in the peripheral blood, the growth/differentiation factor-15 (GDF-15), a member of the transforming growth factor β superfamily.^17^

To investigate the relationship between neuronal, glial and mitochondrial damage with respect to LHON and ADOA optic nerve structural modifications, we measured serum levels of NfL (sNfL), GFAP (sGFAP) and GDF-15 (sGDF-15) in 9 genetically confirmed ADOA and 12 *(11778/14484)* confirmed LHON patients. Biomarkers were evaluated in the context of the clinical impairment and optical coherence tomography (OCT) findings.

Our results suggest that biomarkers reflecting the evolution of neuroaxonal and glial involvement in chronic phases of LHON and ADOA, may be useful for monitoring disease progression and the effects of potential treatments in both preclinical and clinical settings: a particularly important implication for LHON patients for whom, approved treatments are available.

## Materials and methods

### Participants

In this cross-sectional case-control study, 9 ADOA patients with genetically confirmed *OPA1* variants, 12 LHON patients with genetically confirmed 11,778/14,484 mt variants and 40 age- and sex-matched healthy control subjects (HCs) were recruited at the Neurophthalmological Unit of the University of Siena. Genetic analysis was performed in two centers (Siena and Rome).

All patients received clinical and genetic diagnosis from 5 to 10 years before enrolment. They were all taking antioxidants since the diagnosis including high doses of idebenon (900 mg/daily) in two LHON patients, lowering the high dose, after 1 year due to absent visual improvement.

Controls were free from other treatments affecting ocular or neurological functions.

Weight and height were measured in all participants and body mass index was calculated.

After providing a signed informed consent, all subjects underwent the same protocol, including a complete clinical, neurological and neuro-ophthalmologic examination.

Patients were required to have a brain magnetic resonance imaging (MRI) performed no later than 2 years before the study. To minimize the risk that sNfL, sGFAP and sGDF-15 could come from other degenerative processes, only patients with normal MRI beside optic atrophy, and normal neurological examination were enrolled.

Retinal nerve fibre layer (RNFL) thickness OCT scans were acquired in 9 ADOA patients, 11 LHON patients and 34 HC subjects. Ganglion cell layer (GCL) OCT scans were acquired in 8 ADOA patients, 11 LHON patients and 34 HC subjects at the same day of the clinical examination and the acquisition of the blood sample for sNfL, sGFAP and sGDF-15 assay.

The study was performed according to the criteria of the Declaration of Helsinki, and it was approved by the local Ethic Committee of the Azienda Ospedaliera Universitaria Senese, EVAlab protocol CEL no. 48/2018.

### Neuro-ophthalmological examination

All subjects received a complete neuro-ophthalmological examination including neurological examination and best-corrected visual acuity (BCVA), color sensitivity test using Ishihara plates, intraocular pressure measurement by applanation tonometry, fundoscopy with assessment of the optic disk neurorim shape, color, vasculature and disk cupping. BCVA was assessed using Snellen digital charts and converted from Standard Snellen notation to logarithm of the minimal angle of resolution (logMAR) values.

### Optical coherence tomography (OCT): retinal fibers layers (RNFL) thickness and ganglion cells layers (GCL) scans

Spectral-domain optical coherence tomography (SD-OCT) examinations was performed using the Spectralis HRA+OCT (Heidelberg Engineering, Heidelberg, Germany) device. The SD-OCT protocol encompassed the computation of RNFL thickness in the peripapillary region and the GCL thickness in the macula (where the GCL is defined as the thickness from the inner boundary of the GCL to the outer boundary of the Inner Plexiform Layer).

After pupil dilation, imaging was performed by the same operator for all patients.

A circle scan was manually focused on the optic disc to obtain the peripapillary RNFL thickness. Scans were taken using the high-speed mode and automatic real time averaging 62 B-scan (ART = 62) frames to improve image quality. A volume scan consisting of 61 B-scans with an interscan distance of 123 μm, covering a 30° × 25° square fixed on the fovea was obtained to measure the macular GC layer thickness. Scans were obtained with the high-speed mode, using ART = 10. RNFL and GC layer thicknesses were automatically calculated by the built-in software of the device. The inner limiting membrane and the GCLs were used as segmentation boundaries to calculate both global and sectorial (temporal-superior, temporal, temporal-inferior, nasal-superior, nasal and nasal-inferior) peripapillary RNFL thickness values. GCL thickness values were calculated using the RNFL and GC layer as segmentation boundaries and reported based on an Early Treatment Diabetic Retinopathy Study (ETDRS) grid with circular diameters of 1, 3 and 6 mm, centered on the fovea. Manual segmentation of SD-OCT B-scans was performed in cases in which the automated segmentation failed.

Additionally, OCT measures of sex and age matched controls were selected from the healthy control subjects.

### Genetic procedure

DNA was extracted from blood samples using QIAamp DNA blood kit (Qiagen, Limburg, Netherlands). Genetic analysis of *MT-ND1*, *MT-ND4*, *MT-ND6* and *OPA1* genes was performed by PCR using specific primers (available upon request). Following purification of the PCR products using QIAquick PCR Purification kits (Qiagen), Sanger sequencing was performed using the ABI 3500 automated sequencer (Applied Biosystems, Foster City, CA). The results were analyzed using Chromas (version 2.33) software and compared with reference sequences: NM_130837.2 (*OPA1* transcript variant 8) and Revised Cambridge Reference Sequence (rCRS) of the Human Mitochondrial DNA (GenBank sequence NC_012920). The American College of Medical Genetics and Genomics (ACMG) criteria were used to classify detected variants.

### Biochemical procedure and sNFL, sGFAP and sGDF-15 assay

Blood samples from 9 ADOA patients, 12 LHON patients and 40 HCs were collected and sNfL, sGFAP and sGDF15 were assessed. sNfL and sGFAP concentrations were measured in each subject’s sample using the commercially available immunoassay kits for NfL and GFAP – SimoaTM assay Neurology 2-Plex B (GFAP, NfL) Assay Kit (Catalog #103520; Quanterix, Billerica, MA, USA) which runs on the semi-automated ultrasensitive SR-X Biomarker Detection System (Quanterix). Samples were diluted at 1:4 and randomly distributed on 96-well plates. Quality control (QC) samples provided with the kit had concentrations within the predefined range and the coefficient of variance across the plates was maintained below 10%. All samples were analyzed blindly under alpha-numeric codes. The diagnostic codes were broken only after QC-verified NfL and GFAP concentrations were reported to the database manager.

sGDF-15 was assessed in each patient’s and HCs’ sample using GDF-15 Human ELISA kit (Bio-Techne, USA R&D Systems, Inc.), read on iMark Absorbance Microplate Reader (Bio-Rad), according to the manufacturer’s instructions.

sNFL, sGFAP and sGDF-15 values were corrected for age and BMI in each subject.

All the biochemical analyses were performed at the laboratory of the Centre of Precision and Translation Medicine, University of Siena, Italy.

### Statistical analysis

Data were summarized as number of patients (percentage/frequency) and median (25th–75th percentiles). Group differences were assessed using the analysis of covariance. To avoid interocular bias, the average values of the two eyes for each subject were calculated. In patients, quantitative visual BCVA, OCT and biochemical data were compared with the Fisher exact test.

Shapiro-Wilk and Bartlett test statistics were employed to investigate the normality and homoscedasticity of the data samples.

sNFL, sGFAP and sGDF-15 values were skewed. Therefore, sNFL, sGFAP and sGDF-15 data values were log 10 transformed. To examine differences between the three groups, analysis of covariance was performed with log 10 sNfL, log 10 sGFAP and log 10 sGDF-15 levels as dependent variables and the ADOA, LHON and HCs groups as fixed variables. Age and body mass index (BMI) were used as covariates for log 10 sNfL, log 10 sGFAP analyses, whereas only age was adopted as covariate for log 10 sGDF-15 analysis. The importance of BMI and age, particularly when analysing sNfL concentrations, has been previously highlighted.^18^ Conversely, only modest elevations in sGDF-15 concentrations are age related.^19^ Tukey post-hoc test was finally applied to assess exactly which groups differ from each other.

The variance of the mean quadrant macular GCL thickness and the mean quadrant RNFL thickness values were different between groups. Therefore, Welch Anova and post-hoc Games-Howell test statistics were used to compare the mean quadrant macular GCL thickness and the mean quadrant RNFL thickness values of ADOA, LHON and HC groups.

Due to the absence of neurodegeneration marker assessments in the OCT control group, we conducted the correlation analysis between OCT data and blood markers exclusively within the patient groups. This approach enabled the utilization of complete data sets, thereby ensuring accurate and unbiased correlation analysis. Non-parametric partial correlation (Spearman correlation) with age as control variable was performed for the analysis of statistical dependence between OCT variables, BCVA and biochemical variables: sNFL, sGFAP and sGDF-15 in LHON and ADOA subjects.

The value of *P* < 0.05 was considered statistically significant. Analysis results and graphs were generated with Jamovi software (Version 2.3.28.0) and Matlab Statistical Toolbox (Version 2019b).

## Results

### Clinical, demographic and genetic characteristics

Demographic and clinical features of ADOA and LHON patients and HC are summarized in Table 1.

**Table 1:** Demographic features of patients with autosomal dominant optic atrophy (ADOA) and Leber hereditary optic neuropathy (LHON) included in the study

Patients	Pathology	Mutation (OPA1: NM_130837.3; MT-ND1, MT-ND4, MT-ND6: GenBank sequence NC_012920)	Sex	Age	Visual acuity (logMar)
1	ADOA	OPA1: c.2936_2938del; p. (Lys979del)	F	46	0.10
2	ADOA	OPA1: c.2936_2938del; p. (Lys979del)	F	64	0.20
3	ADOA	OPA1: c.2936_2938del; p. (Lys979del)	M	16	0.50
4	ADOA	OPA1: c.2936_2938del; p. (Lys979del)	M	10	0.20
5	ADOA	OPA1: c.2936_2938del; p. (Lys979del)	F	37	0
6	ADOA	OPA1: c.2873_2876del; p. (Val958Glyfs*3)	M	36	0.40
7	ADOA	OPA1: c.2873_2876del; p. (Val958Glyfs*3)	M	69	1.30
8	ADOA	OPA1: c.428T>C; (p.Ile143Thr)	F	/	1.60
9	ADOA	OPA1: c.333insG (p.Gly112Trpfs*9)	M	48	0.20
10	LHON	ND6: m.14484T>C	M	49	1.60
11	LHON	ND4: m.11778G>A	F	66	1.30
12	LHON	ND4: m.11778G>A	M	51	1.30
13	LHON	ND4: m.11778G>A	M	46	0
14	LHON	ND4: m.11778G>A	F	21	0
15	LHON	ND4: m.11778G>A	F	48	0
16	LHON	ND4: m.11778G>A	M	70	1.30
17	LHON	ND4: m.11778G>A	M	62	1.30
18	LHON	ND6: m.14484T>C	F	43	1.30
19	LHON	ND4: m.11778G>A	F	51	0.70
20	LHON	ND6: m.14484T>C	M	21	0.10
21	LHON	ND6: m.14484T>C	F	36	/

Age, gender and BMI did not significantly differ across ADOA, LHON and HCs: age – ADOA: median, 46 years (36–63); LHON: median, 48.5 years (41.3–53.8), HC: 48.5 years (38–63); gender – ADOA (55.5%, male), LHON patients (50%, male) and HCs (50%, male); and BMI – ADOA: median, 23 kg/m^2^ (23–24); LHON: median, 24.1 kg/m^2^ (23.8–26.4); and HCs: median, 25 kg/m^2^ (24–25).

**Figure 1:**
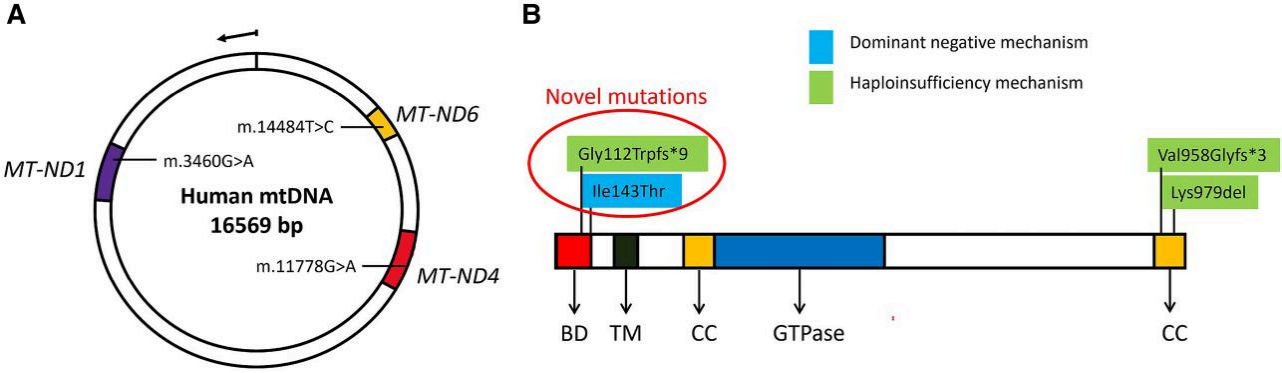
**Mitochondrial genome and OPA1 variants.** (**A**) Mitochondrial genome (mtDNA). The three common primary variants are at mitochondrial nucleotide positions mt.3460, mt.11778, and mt.14484. The variants affect *MT-ND1*, *MT-ND4*, and *MT-ND6* subunit genes of complex I, respectively. (**B**) Distribution of *OPA1* variants observed in our cohort. Each distinct variant observed in our cohort is represented above the gene: four patients had the in-frame deletion p.Lys979del, two patients had the frameshift p.Val958Glyfs*3, and two patients with the missense p.Ile143Thr and frameshift p.Gly112Trpfs*9 variant, respectively; the last two mutations are novel. The in-frame deletions and the frameshift variants are *OPA1* variants predicted to lead to haploinsufficiency; instead, missense variants are associated with a dominant negative mechanism. *OPA1* contains a basic domain (BD), a predicted transmembrane region (TM), two coiled-coil regions (CC), and the GTPase domain (GTPase).

A primary LHON mtDNA variant was present in 12 patients (Fig. 1). The m.11778G>A variant was found in eight patients (66.6% of the LHON cases), and the m.14484T>C variant in four patients (33.3% of the LHON cases). Nine ADOA patients shared four heterozygous *OPA1* variants: four patients the c.2936_2938del (p. Lys979del) variant,^20^ two patients the c.2873_2876del (p. Val958Glyfs*3) variant^10^ and two single patients the novel variants c.428T>C (p.Ile143Thr) and c.332dup (p.Gly112Trpfs*9). The missense Ile143Thr variant has been previously proved to induce a dominant negative effect while the other variants are predicted to lead haploinsufficiency.^21^

### Visual acuity data

The average visual acuity of ADOA patients was 0.7 LogMAR (LogMAR 1.60-0), while the average visual acuity of the LHON patients was 0.9 LogMAR (r 1.60-0 LogMAR). All patients exhibited visual impairments for a duration exceeding 3 years without any recovery.

### OCT characteristics

**Figure 2:**
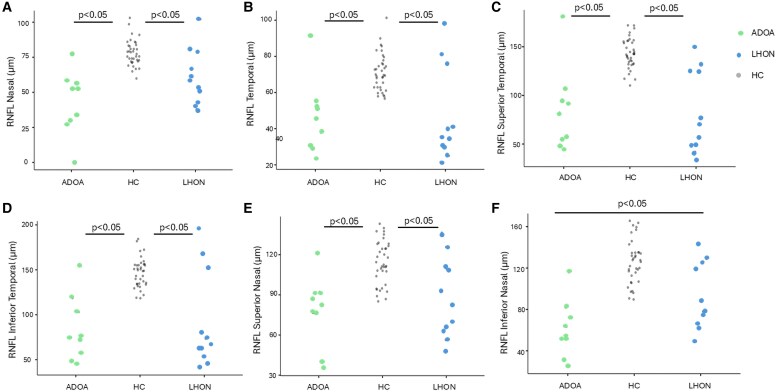
**Data points.** RNFL (retinal nerve fiber layer) of Nasal (**A**), Temporal (**B**), Superior Temporal (**C**), Inferior Temporal (**D**), Superior Nasal (**E**), Inferior Nasal (**F**) quadrants in ADOA (Autosomal Dominant Optic Atrophy) patients (*n* = 9), LHON (Leber Hereditary Optic Neuropathy) patients (*n* = 11), and Healty Controls subjects (HC) (*n* = 34); statistical differences (Games Howell test, *P* < 0.05) are also reported.

**Figure 3:**
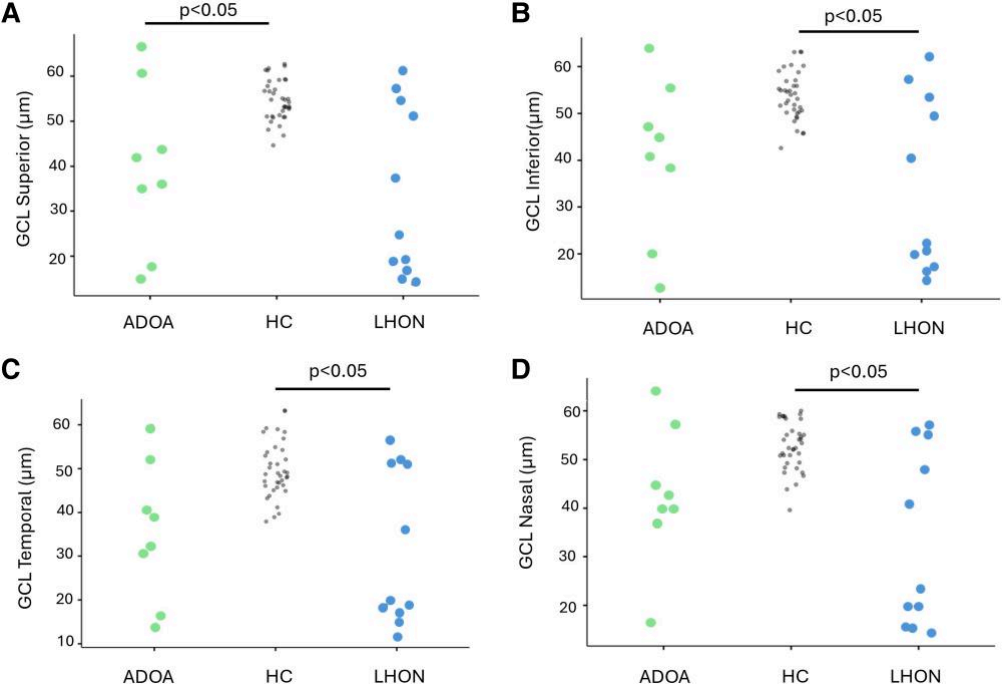
**Data points.** GCL (Ganglion Cells Layers) of Nasal (**A**), Superior (**B**), Temporal (**C**), and Inferior (**D**) quadrants in ADOA patients (*n* = 8), LHON (*n* = 11), and HC subjects (*n* = 34). Single data points representing the observations for each of the ADOA and LHON patients and HC participants; statistical differences (Games Howell test, *P* < 0.05) are also reported.

No significant correlation was found between OCT parameters and age (Table 2 and Figs. 2 and 3).

**Table 2:** Median and (25th–75th) percentiles values of OCT data

	ADOA	LHON	HCs
	Median (25th–75th percentiles)	Median (25th–75th percentiles)	Median (25th–75th percentiles)
Temporal RNFL (µm)	46.0 (30.3–53.6)	35.5 (30.3–67.3)	69.8 (63.0–78.0)
Superior Temporal RNFL (µm)	82.0 (53.5–98.3)	71.0(49.6–125.0)	142.3(133.0–156.0)
Inferior Temporal RNFL (µm)	74.5 (55.4–106.9)	67.5 (56.5–135.1)	149.3 (134.0–156.0)
Nasal RNFL (µm)	52.5 (29.3–57.4)	58.0(44.4–75.5)	78.0 (72.0–83.5)
Superior Nasal RNFL (µm)	83.0 (67.4–91.6)	82.0 (63.9–109.9)	113.5 (97.5–128.0)
Inferior Nasal RNFL (µm)	54.5 (47.4–74.1)	78.5 (68.8–124.3)	126.8 (107.0–135.0)
Superior GCL (µm)	39.0 (26.0–51.5)	25.0 (17.3–53.3)	53.0 (51.0–57.0)
Inferior GCL (µm)	43.0 (29.0–51.5)	22.0 (17.8–53.0)	53.0 (50.0–56.0)
Temporal GCL (µm)	35.5 (23.5–46.5)	20.0 (17.5–51.0)	48.0 (46.0–52.0)
Nasal GCL (µm)	41.5 (38.5–50.5)	23.0 (16.3–53.3)	52.0 (49.0–55.0)

Median and (25th–75th) percentiles values of the mean macular GCs thickness and of the mean RNFL thickness values for each quadrant of ADOA, LHON patients, and HCs.

The mean macular GCL and peripapillary RNFL thickness values in ADOA patients was 39.5 µm and 66.2 µm, respectively. The mean macular GC layer and peripapillary RNFL thickness values in LHON patients was 33 µm and 77.9 µm, respectively. The mean macular GCL and peripapillary RNFL thickness values HCs was 51.5 µm and 107.5 µm, respectively. The median and 25th–75th percentile values of the mean GCL thickness and of the mean RNFL thickness values for each quadrant of ADOA and LHON along with those of HCs are reported in Table 2.

ADOA and LHON patients had significantly lower values of RNFL thickness than HCs. Moreover, for the inferior nasal RNFL thickness, ADOA patients showed significantly (*P* = 0.00085676) lower values than LHON patients (Fig. 2).

LHON patients showed values of GCL thickness significantly lower than HCs. No significant differences were observed between ADOA and LHON (Fig. 3).

### sNfL, sGFAP and sGDT-15 profile: LHON versus ADOA versus HCs

**Figure 4:**
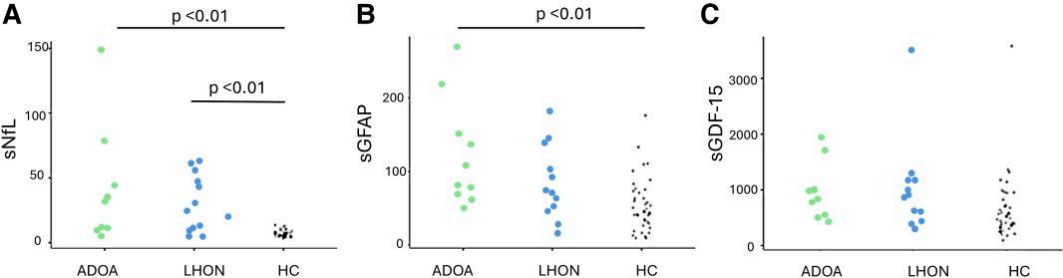
**Data points.** Serum concentrations of neurofilament light chain (sNfL) (**A**), glial fibrillary acidic protein (sGFAp) (**B**), and growth/differentiation factor-15 (GDF-15) (**C**) in autosomal dominant optic atrophy (ADOA) (*n* = 9), Leber hereditary optic neuropathy (LHON) (*n* = 12), and healthy controls (HCs) (*n* = 40). Single data points (grey dots) representing the observations for each of the ADOA and LHON patients and healthy control participants are also reported together with the statistical differences (Ptukey test, *P* < 0.05).

Age- and BMI-corrected sNfL levels were increased in ADOA patients (median sNfL 31.4; 10.3–42.6) and LHON patients (median sNfL 27.3; 11.4–47.9) compared to HCs (median sNfL 6.71, 5.11–8.75; *P* < 0.01 for both) (Table 3 and Fig. 4). No significant difference was found by comparing ADOA and LHON patients.

**Table 3:** Median and (25th–75th) percentiles of fluid biomarkers

	ADOA	LHON	HCs
	Median (25th –75th percentile)	Median (25th- –75th percentile)	Median (25th–75th percentile)
sNfL (pg/mL)	31.4 (10.3–42.6)	27.3 (11.4–47.9)	8.9 (5.9–15.7)
sGFAP (pg/mL)	77.3 (68.3–134.6)	72.8 (50.7–111.0)	52.4 (31.8–84.9)
sGDF-15 (pg/mL)	804.0 (504.0–984.0)	896.0 (557.0–1172.0)	516.0 (364.0–776.0)

Median and (25th–75th) percentiles values of the mean serum concentrations of neurofilament light chain (sNfL) (A), glial fibrillary acidic protein (sGFAP) (B), and growth/differentiation factor-15 (GDF-15) in autosomal dominant optic atrophy (ADOA), Leber hereditary optic neuropathy (LHON), and healthy controls (HCs).

Age- and BMI-corrected sGFAP levels were increased in patients with ADOA (median sGFAP 77.3, 68.3–135) and LHON patients (median sGFAP 72.8, 50.7–111) with respect to HCs (median sGFAP 44.1, 28.3–71.7). However, the difference was statistically significant only between ADOA and HCs (*P* < 0.01).

No significant difference was found when age and BMI corrected log sGDF-15 levels were analyzed (LHON: median sGFD-15 896 pg/ml, 557–1173; ADOA median sGDF-15 804 pg/ml, 504–984; HCs: median sGDF-15 516 pg/ml, 359–750 (see Table 3 and Fig. 4).

**Figure 5:**
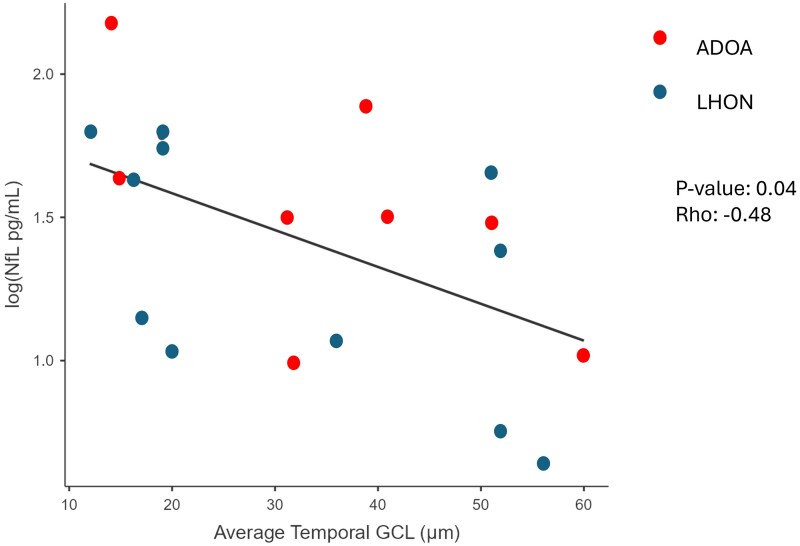
**Correlation between CGs data and log10 sNfL.** Partial correlation between Log10 levels of serum neurofilament light chain (sNfL) and retinal ganglion cell layer thickness measured with OCT, with age as control variable, in autosomal dominant optic atrophy (ADOA) (*n* = 9), and Leber hereditary optic neuropathy (LHON) (*n* = 12). *P* and rho values obtained by the Spearman correlation test are indicated in the legend.

sNfL levels showed a negative correlation with average temporal GCL OCT values (R = −0.48; *P* 0.04) in both patient’s groups (Fig. 5).

No other significant correlation nor significant difference was found among levels of sGFAP and sGDF-15 and OCT parameters and BCVA in all groups.

## Discussion

Our cross-sectional study of patients with chronic optic neuropathy due to ADOA and LHON shows a different OCT pattern of GCL and RNFL thickness, with GCL more severely affected in LHON compared to ADOA and RNFL significantly thinner nasally in ADOA than in LHON.

Furthermore, both groups exhibited increased levels of sNfL which correlated with the average reduction of temporal GCL thickness. sGFAP level increased in all patients, although reaching statistical significance only in ADOA patients compared to controls. The elevation of sGFAP in both conditions suggests glial involvement, which appears to be more pronounced in ADOA.

Although sNfL and sGFAP are not specific to optic nerve atrophy or visual pathway degeneration and may indicate neurodegeneration in other areas of the central nervous system (a possibility encompassed in the well-described ADOA+ and LHON+ phenotypes), this is unlikely in our patient cohort, who were asymptomatic for non-visual neurological symptoms and had normal brain MRI findings apart from optic atrophy. Nevertheless, the possibility of subclinical involvement beyond the visual pathways cannot be excluded.

Finally, serum levels of GDF-15 – a cytokine considered a peripheral marker of mitochondrial myopathies – were elevated in both patient groups compared to controls, without any significant difference between the two.

### sNFL increase and related mechanisms of neurodegeneration in LHON and ADOA

Despite a final common pathological pathway, ADOA and LHON are clinically distinct. Neurodegeneration differs principally for the timing (chronic in ADOA versus acute in LHON) and the axonal damage propagation. Indeed, none of the preclinical signs of LHON, i.e the axonal swelling and the vascular changes, is reported in ADOA. Furthermore, while LHON is a sequential, apparently synchronized and dramatic wave of cell death which propagates circumferentially from the infero-temporal segment of the optic disc to the remaining quadrants,^22^ in ADOA, the process is slower, starts in childhood bilaterally and symmetrically and might have different pattern of propagation during the disease course.

Histopathological studies fail to distinguish two diseases based on the pathological changes: post-mortem optic nerve specimens from patients with ADOA and LHON show a similar massive loss of parvocellular retinal GC axons, especially the smaller ones replaced by reactive gliosis, with a variable preservation of large axons, including those of the melanopsin pathway.^1,23,24^ Electron microscopy reveals in both conditions, patchy of supernumerary small mitochondria with disorganized cristae, mostly concentrated in the prelaminar portion of the optic nerve: this increased mitochondrial biomass is a reaction to impaired ATP production, but its efficacy is limited.

Here we propose that this defective mitochondrial homeostasis may result in GCL and RNFL thinning in LHON and ADOA, as supported by OCT findings and elevated sNfL.

NfL is a key component of the neuronal and axonal cytoskeleton involved in mechanical stabilization, axonal transport and mitochondrial anchoring – processes regulated by mitochondrial membrane potential and NfL phosphorylation. All three NfL subtypes (light, intermediate and heavy chains) are expressed in human retinal GCs and released into the extracellular space following neuroaxonal injury. From the interstitial fluid it diffuses into adjacent body fluids including CSF, blood, vitreous and aqueous.^25-27^

sNfL has recently been validated as body fluid biomarker of neuro-axonal damage in different acute or chronic neurological disorders with a linear relationship demonstrated in vitro between the number of degenerating neurons and the level of serum NfLs.^15^

The changes in blood sNfL levels observed in our patients resemble those reported in multiple sclerosis,^28^ where sNfL levels appear to correlate with retinal neuroaxonal loss. However, sNfL levels are not disease specific and they increase in different systemic diseases (e.g. diabetes or kidney and heart failure) in the context of glymphatic and CSF fluid dynamic changes or in physiological conditions including normal aging.^29^ Elevation of NfL concentrations has also been found in the anterior chamber fluid, but not in the serum, of patients with glaucoma.^30^

With respect of optic neuropthaies, high levels of phosphorylated neurofilaments heavy chain (pNF-H) have been reported in affected and carrier individuals of a large Brazilian LHON pedigree, supporting the link between blood NfL increase and neurodegeneration in the disease.^31^

Even if serum NfLs indicates a general and nonspecific neuroaxonal damage, its elevation in our cohort suggests that the neuronal loss in LHON and ADOA, while primarily compartmental, may be sufficiently extensive – along or beyond the visual pathway – to be reflected in peripheral blood.

In our study, sNfL levels inversely correlated with the GCL thickness, particularly in the temporal quadrant, the thinner perifoveal sector in both groups (Fig. 3). Elevated sNFL could thus reflect different mechanisms of mitochondrial-related damage of GC affecting the axons but also the soma of these cells.^6^ Preclinical models show in both diseases a convergence to an OXPHOS defect, leading to leaky mitochondrial membrane potential, disorganization of the cristae, increased ROS toxicity, specifically superoxide and release of cytochrome-c, followed by caspase-dependent apoptotic nuclear events.^32-40^ Notably, a caspase negative apoptosis has been identified in LHON cybrids suggesting that other apoptotic factors (i.e. endonucleases), can be released in the cytosol by damaged mitochondria, thus expanding the possible pathways of mitochondria related cell death.^35^

Increased autophagy, another pro-apoptotic pathway, may contribute to GCs death in both diseases.^41,42^ Supporting this hypothesis, dysregulation of basal autophagy or mitophagy exceeding the compensatory effects of mitobiogenesis, has been reported in various cellular models of ADOA and LHON^43-47^ as well as in glaucoma.

Excessive mitophagy has harmful effects on the neurons of patients with *OPA 1* variants, particularly at the level of axonal hillock-initial segments, where mitochondrial trafficking is altered, and axons are depleted of these organelles so that the GCs soma are stuffed by autophagosome aggregates.^48^ This region, where the action potential is generated, contains a complex network of microtubules and NfLs. The susceptibility to damage at that level, therefore, may trigger NfLs desegregation explaining the rising of NfL. Furthermore, the decline of mitochondrial membrane potential, consequent to OXPHOS deficit, might result in the loss of the mitochondria–NfL interaction with subsequent dysregulation of mitochondrial distribution that further contributes to axonal injury and NfL disintegration.

Similar findings have recently been observed in cells and cybrids of LHON-affected individuals where excessive mitophagy, in the soma of symptomatic subjects, is opposed to efficient compensatory biogenesis observed in LHON carriers, suggesting a role for the residual compensatory mitobiogenesis in modulating the clinical expression.^49^

However, other elements may account for a differently mediated GCL death to explain the distinct presentation of the two diseases. Glutamate excitotoxicity or the spreading of small apoptotic molecules via axonal gap junctions from GCs to neighboring cells have been proposed.^3^

Here we suggest a direct modulatory effect of the glia, as indicated by the increased level of GFAP.

### sGFAP and the possible involvement of retinal astrocytes in ganglion cell damage

Our study documented a significant increase in sGFAP level in ADOA patients, and even if less significantly, sGFAP was also increased in LHON patients, suggesting a substantial activation of astrocytes in both diseases.

Retinal astrocytes have shown to become reactive in response to traumatic injury, hypoxia or glaucoma, modifying functions and gene expression (such as upregulating GFAP and phagocytic genes) and losing their typical arrangement to culminate towards gliosis.^50,51^

In this regard, previous studies on animal models, have shown that *OPA1* gene variants causing loss of GCs, was accompanied by activation of astrocytes.^52,53^ Also in LHON patients, the mitochondrial complex I dysfunction in the retina has been shown to trigger an innate immune and inflammatory response that in addition to the loss of retinal GC, results in astrocyte activation.^54^

In parallel, the role of optic nerve head (ONH) astrocytes, in response to optic nerve injury, is an increasingly well studied topic in glaucoma research: similarly, to mitochondria related optic neuropathy, disrupted mitochondrial homeostasis and increased oxidative stress is emerging as a possible causative role of neuroaxonal degeneration in glaucoma.^55,56^

Notably, a few concepts have emerged in this context, that may impact mitochondria related optic nerve damage and glial activation: at the level of the ONH, a peculiar subpopulation of extensively interconnected and morphologically well-defined astrocytes has been identified. Their functional characteristics seem to differ from other retina and optic nerve glial cells.^57^ These astrocytes are actively involved in axonal mitochondria turnover and recycling. Unlike brain glia and retinal Muller cells, which use glycolysis and store glucose in glycogen for energy provisioning, the ONH astrocytes, have a prevalent oxidative phosphorylation energy metabolism, by which they provide greater energy substrates to stressed GCs axons. On the other hands, they may also contribute to increasing the level of ROS and oxidative stress in damaged ONH environment.^57,58^ ONH astrocytes control axon’s survival as documented by their repertoire of antioxidants, including glutathione, catalase and superoxide dismutase pathways, protecting at that level, axons against oxidative injury.^58^

Moreover, the ONH astrocytes represent a specific subpopulation of glial cells, morphologically and transcriptionally different from brain astrocytes with a peculiar functional specialization.^59^

In the retina, astrocytes show two morphological categories, one with a stellate shape, contacting the vasculature in the GCL and another with elongated processes that run parallel to axons, in the nerve fibres layer. In the lamina cribrosa within the nerve head, axons are ensheathed by a dense meshwork of GFAP-positive and Acquaporine4 (APQ4) negative astrocytes, forming a glial lamina extensively connected to the vascular network.^57,58^ The tight link with vasculature allows the astrocytes to extract metabolites glucose and oxygen from the blood, which are useful energy substrates for axons activity and survival.^59^

Another aspect of retinal astrocytes is that they are extensively networked across anatomically long distances, including contralateral eye, by gap-junctions, suggesting a role of this network in the redistribution of metabolic resources in conditions of metabolic stress, such those occurring in the mitochondrial-related-ONH diseases. Recently it has been shown that the interconnected astrocyte network can at least temporarily reduce the vulnerability of stressed optic nerves through glycogen mobilization and a long-range energy provisioning.^58,60^

A further characteristic of ONH astrocytes is their activity of degradation of axonal mitochondria by trans-mitophagy, a process in which mitochondria are extruded by axons before being engulfed and eliminated by astrocytes. The astrocyte trans-mitophagy capacity is estimated to exceed mitophagy by GCs in the GCL.^61^ Intriguingly, the astrocytes of the ONH express elevated levels of genes related to mitochondrial protein translation and oxidative phosphorylation. This could imply that these astrocytes may transfer protein to axons supporting mitobiogenesis in conditions of high energy demand.^62,63^

Taken together, these data indicate a role for ONH astrocytes in sustaining GC axons to preserve mitochondrial homeostasis and protect ONH environment from ROS toxicity. However, in the case of damaged optic nerves, when ROS production exceeds their antioxidant capacity, they might also accelerate rapid axonal damage.

In such a scenario, the interconnected astrocyte network could increase the vulnerability of stressed optic nerves through mobilization and transfer to the contralateral optic nerve, of free radicals or catabolic enzymes, or toxic molecules, triggering the massive propagation of the optic nerve damage as seen in acute LHON.

This metabolically active astrocyte network could also explain the bilateral improvement of visual acuity after one eye intravitreal injection of *rAAV2/2-ND4* (GS010) (recombinant replication-defective adeno-associated virus, serotype 2), in the RESCUE and REVERSE gene therapy trials^64^ or the evidence of *AAV2/ND4* in the post-mortem fellow eye optic nerve.^65^

While pointing toward the involvement of the glia in LHON, the increased level of sGFAP we found in our patients failed to reach significance perhaps because of the small sample size of the chronic stage of our population. Further studies are definitely warranted to prove this hypothesis.

### GDF-15

Unlike NfL and GFAP, we did not identify any significant difference of GDF-15 levels in the three examined groups. GDF-15 is a cytokine distributed in many organs, and it is considered a general biomarker of oxidative stress and inflammation-induced damage in mitochondrial myopathies.^66^ Several studies evidenced increased levels of this factor in the blood of mitochondrial patients, proposing it as a useful biomarker for these pathologies, especially for diseases due to defective mtDNA translation or maintenance. However, elevated GDF-15 levels in blood have been found also in non-mitochondrial myopathies and, more in general, in non-mitochondrial diseases. Moreover, within the CNS, this cytokine has been observed to function as a powerful neurotrophic factor for motor and sensory neurons.^67^

Our data does not support the involvement of GDF-15 in the process leading to GC loss, at least in the chronic stage. Nevertheless, the role of GDF-15 and its significance in the pathogenesis of these diseases require further investigations. In this regard, the finding in animal models that treatment with GDF-15 has neuroprotective and neurotrophic effects on GCs is of great significance, underscoring the potential importance of this cytokine in GC degenerative diseases.^68^

### Different patterns of RNFL and GCs thickness in ADOA and LHON

BCVA did not differ between the two groups of patients.

When compared to controls, we also found a diffuse GCL thinning in all perifoveal quadrants, with the temporal quadrants being the thinnest in both diseases.

Also, although the difference was not statistically significant, patients with LHON tended to show a more generalized GCL loss than those with ADOA, in line with the expected disease pattern (see Fig. 3 and Table 2).^69^

The OCT GCs thickness of the temporal macular sector reduces later in both ADOA and LHON, while the inferior-internal nasal sector, containing the smallest parvocellular GCs whose axons converge to the PMB, is earlier and more severely affected in both diseases.^21,70-72^ In chronic and late stages of both diseases, as in our patient cohort, the GC loss extends to all macular areas,.^73^ In the macula, GCs show higher density and larger caliber in the temporal sector and this could explain the correlation between NfL levels and temporal GCL thickness that we observed in our patients.

Analogously, both groups display a significant reduction of the RNFL thickness with respect to controls, particularly in the temporal quadrants of the peripapillary region according to the prominent involvement of the PMB.^72-75^ However, ADOA patients also showed a reduction of the RNFL in the nasal sectors containing greater caliber axons, with the inferior-nasal quadrants significantly thinner with respect to LHON.

This nasal RNFL involvement in ADOA, along with the more widespread GCL thinning, supports a more diffuse retinal GC degeneration pattern compared to LHON which often spares the nasal sectors even at late stages.^5,72^  ^,76-79^

### Limitations

Although this study cannot definitively establish a direct link between the two serum biomarkers (sNfL and GFAP) and the optic nerve damage, it provides valuable evidence suggesting ongoing neuroaxonal degeneration even in the chronic stages of both LHON and ADOA. The limited sample size, however, restricts our ability to draw firm conclusions about the clinical utility of sNfL and GFAP as biomarkers of neurodegeneration in these optic neuropathies. Additionally, the balanced sex distribution among the LHON patients (50% female and 50% male) may limit the generalizability of our findings to the broader LHON population, which predominantly affects males. Despite these limitations, our results highlight a potential role for these serum biomarkers in capturing disease progression that would need to be confirmed by future studies involving larger cohorts – including pre-symptomatic LHON variant carriers and patients in the acute or early stages of both LHON and ADOA.

## Conclusions

In summary our study investigated clinical, OCT and serum biomarkers (sNfL, GFAP and GDF15) differences in a population of chronic primary mitochondrial optic neuropathies: LHON and ADOA. Our results indicate a more severe loss of GCs and PMB LHON, while ADOA showed a more diffuse a sparser damage of RNFL thinning, including fibers originating from the nasal retina. Since the nasal retina processes input from the temporal visual field and its fibers decussate at the optic chiasm, this raises intriguing questions about potential differences in visual perception and cortical processing between the two conditions.

Moreover, the elevated plasma levels of sGFAP observed in both patient groups suggest a possible association between ONH astrocyte activation and mitochondrial-driven optic nerve degeneration. Nevertheless, further investigation is needed to fully elucidate the significance of the involvement of the optic nerve head glia.

## Data Availability

We declare that all data will be available for consultation, upon specific request to be addressed to the corresponding author rufa@unisi.it
